# Supplementary material for: Accurate Predictions of Molecular Properties of Proteins via Graph Neural Networks and Transfer Learning
Source: J Chem Theory Comput. 2025 Apr 24;21(9):4830–45. doi: 10.1021/acs.jctc.4c01682 (PMC12080100; doi:10.1021/acs.jctc.4c01682)
Supplement: Supplementary file 1 — ct4c01682_si_001.pdf [file ct4c01682_si_001.pdf]

SUPPLEMENTARY INFORMATION

for

Accurate Predictions of Molecular Properties of Proteins via Graph Neural Networks and Transfer Learning

Spencer Wozniak[1], Giacomo Janson[1], and Michael Feig[1,*]

[1]Department of Biochemistry and Molecular Biology, Michigan State University, East Lansing, MI 48824, USA

*Corresponding author
Michael Feig
603 Wilson Road, Room 218 BCH
East Lansing, MI 48824, USA
mfeiglab@gmail.com
+1-517-432-7439

Figures S1-S25
Tables S1-S8
Supplementary References

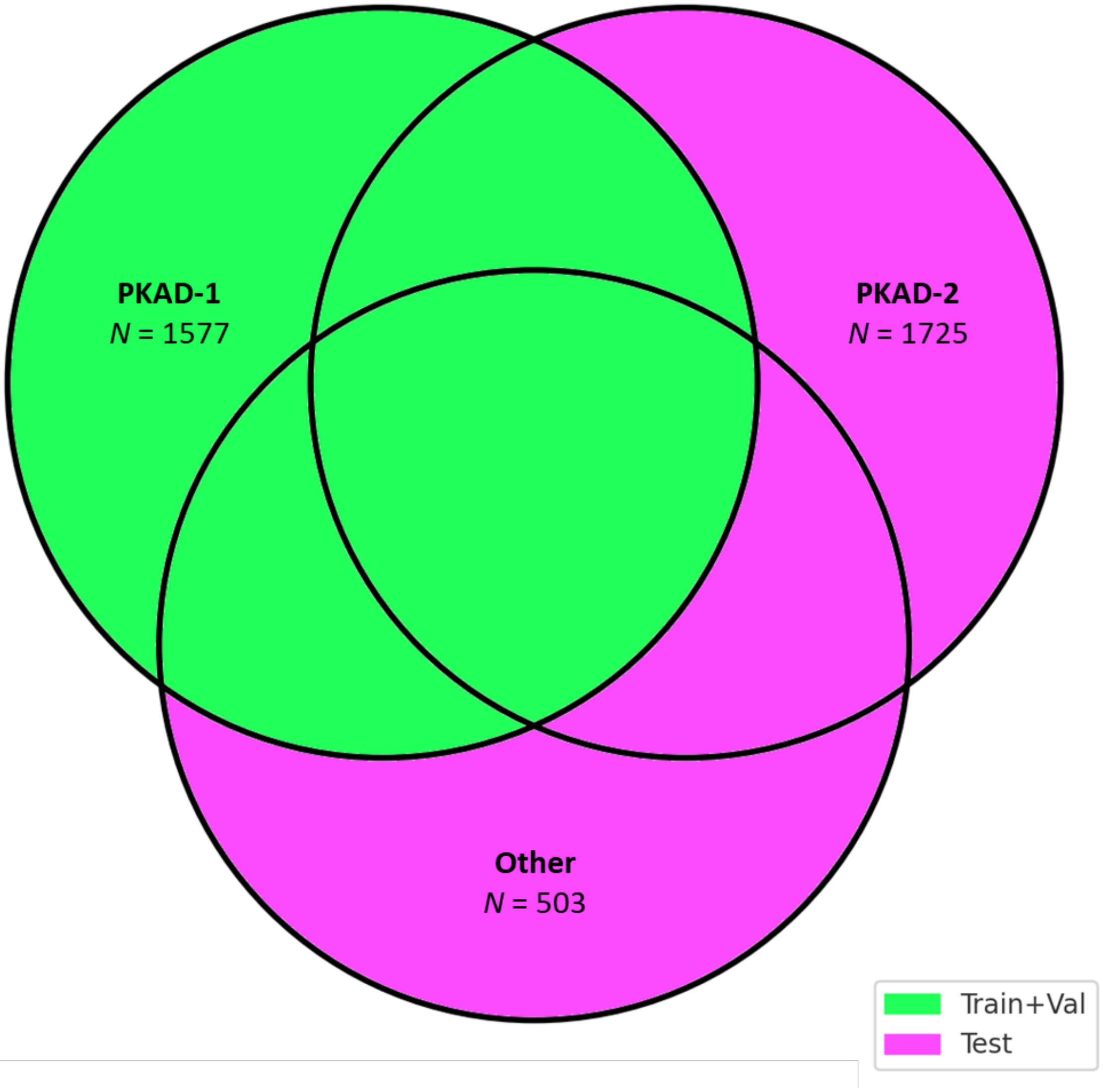

Figure S1. Visual depiction of the sources of our experimental datasets. “Other” includes experimental pK_a data that was obtained from the experimental references listed in Chen *et al*.[1] Gokcan *et al*.[2], and Wilson *et al*.[3] that were not obtained directly from PKAD. Overlap in the Venn diagram represents data points that were considered similar according to the similarity classification strategy outlined in the Methods section. Data points outside of PKAD-1 were only added to the test set if they were dissimilar to data points in the training/validation set according to the similarity classification strategy. *N* represents the total number of data points in each data source.

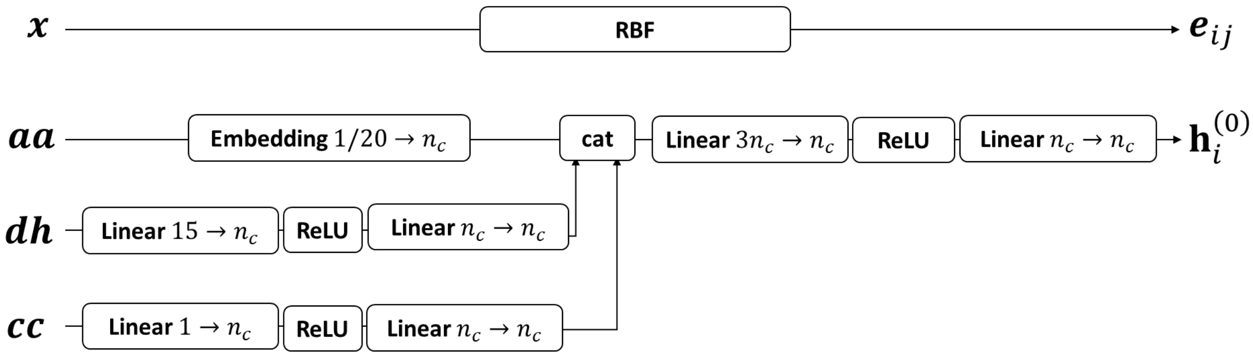

Figure S2. GSnet node and edge embeddings from input features. (n_c: number of hidden channels, RBF: radial basis function, cat: concatenate)

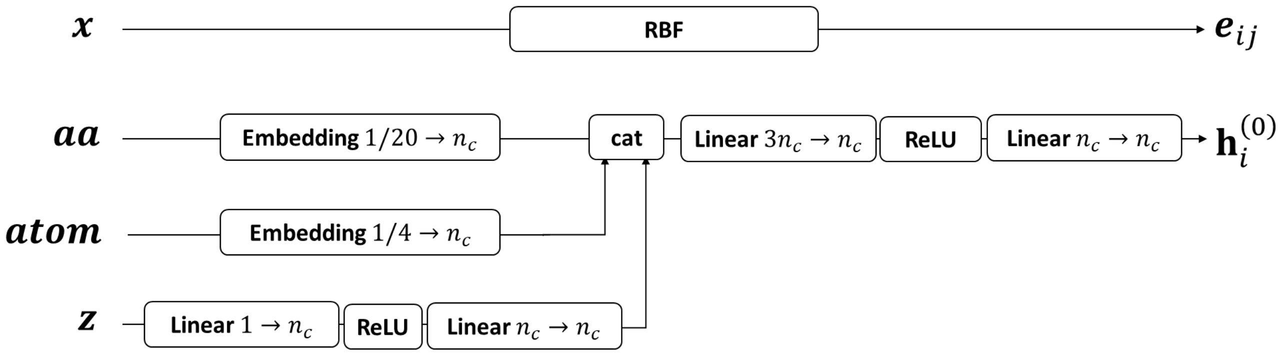

Figure S3. aLCnet node and edge embeddings from input features. (n_c: number of hidden channels, RBF: radial basis function, cat: concatenate, z: charge)

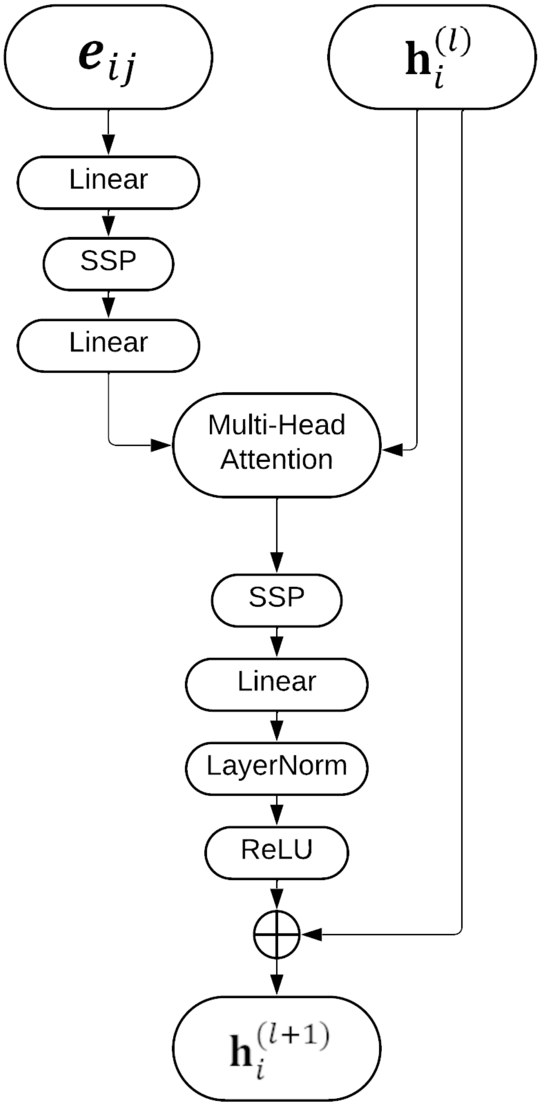

Figure S4. Architecture of message passing blocks in GSnet and aLCnet. $\mathbf{h}_i^{(l)}$ are node features of node i at layer l, $\boldsymbol{e}_{ij}$ are edge features between neighboring nodes i and j, and $\mathbf{h}_i^{(l+1)}$ are node features of node i at the next layer $l+1$. SSP is a ShiftedSoftPlus activation function defined as $\mathrm{SSP}(\mathbf{x}) = ln(\mathbf{1} + e^{\mathbf{x}}) - ln(2)\mathbf{1}$. The operations of the Multi-Head Attention mechanism are described by Shi *et al.*[4]. LayerNorm refers to the layer normalization operation described by Lei Ba *et al.*[5].

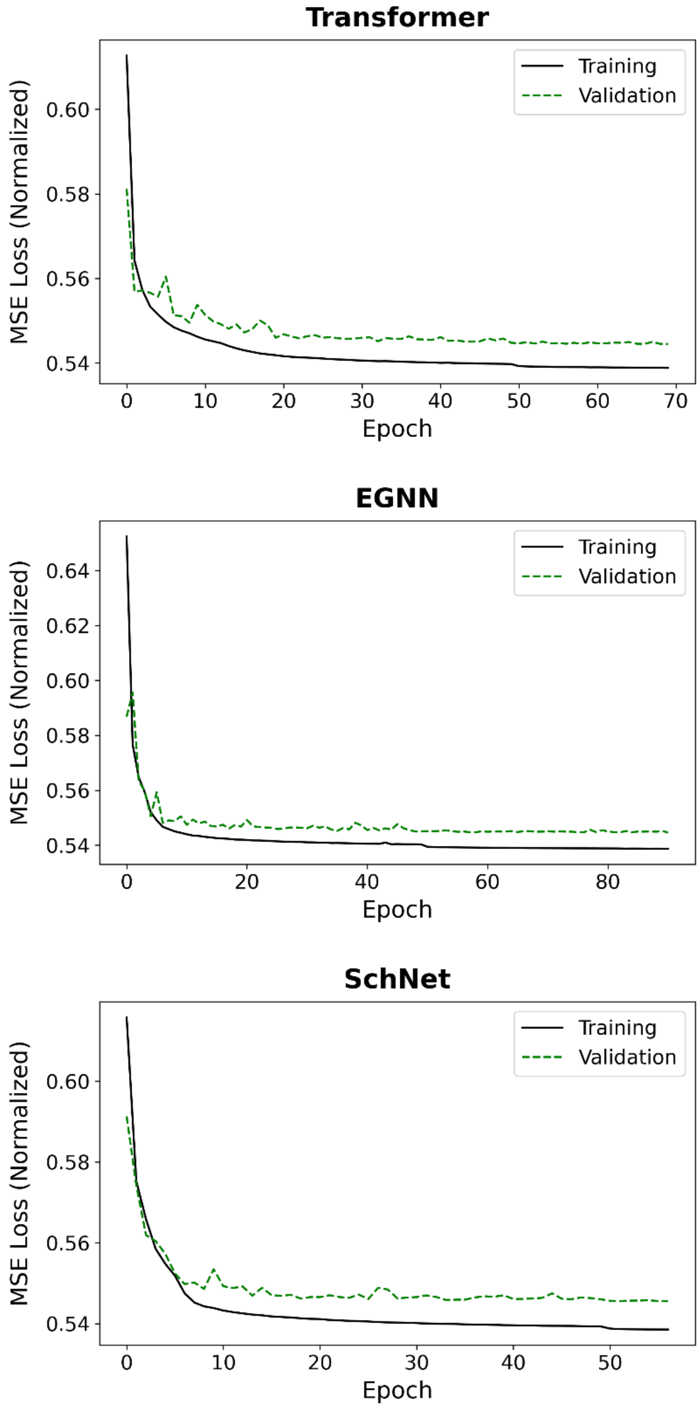

Figure S5. Mean-squared error (MSE) loss across the six target values as a function of epoch during training. Training loss is shown as a solid black line and validation loss is shown as a dashed green line.

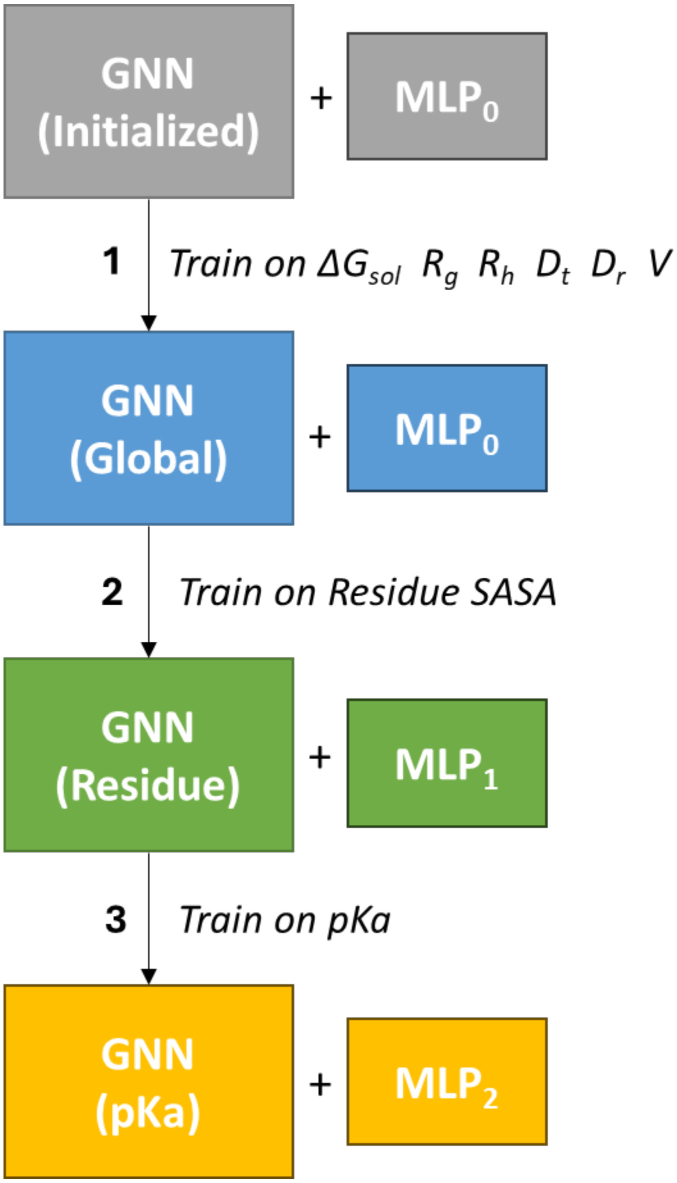

Figure S6. GSnet training scheme for pK_a transfer learning. First, the initialized GNN and MLP were trained on the original target values. Next, this pretrained GNN and a new MLP were further trained to make $SASA_{res}$ predictions. Finally, the GNN as pretrained for $SASA_{res}$ was transferred, and yet another new MLP was introduced and trained to make pK_a predictions. Note that the same process was used for training on both pK_a datasets.

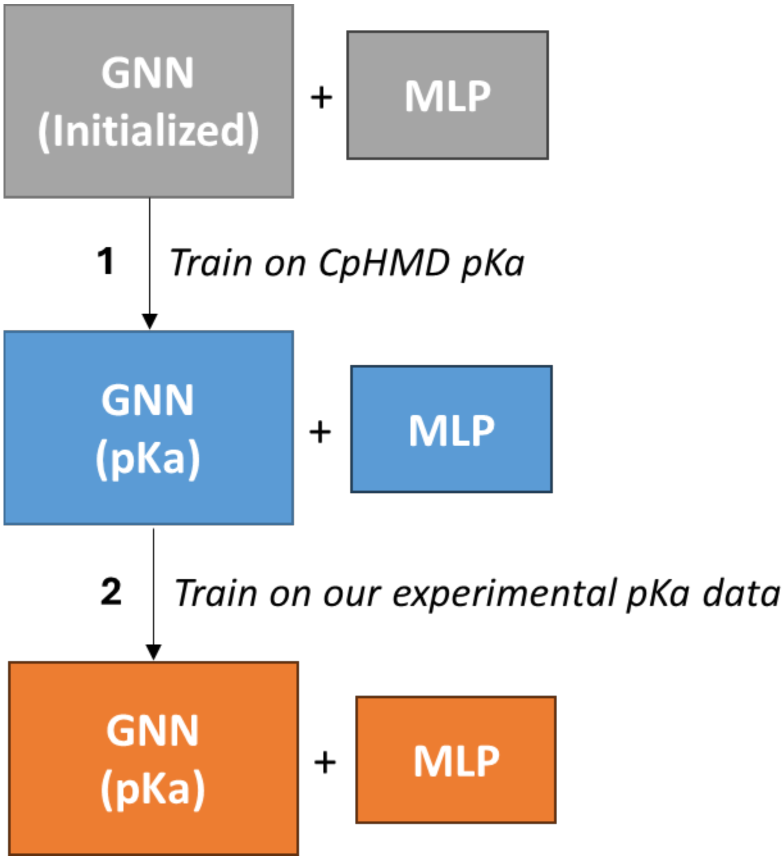

Figure S7. aLCnet training scheme for pK_a transfer learning. First, the initialized GNN and MLP were trained on the simulated pK_a data in the PHMD549 dataset. Next, this pretrained GNN and MLP were further trained on our experimental dataset.

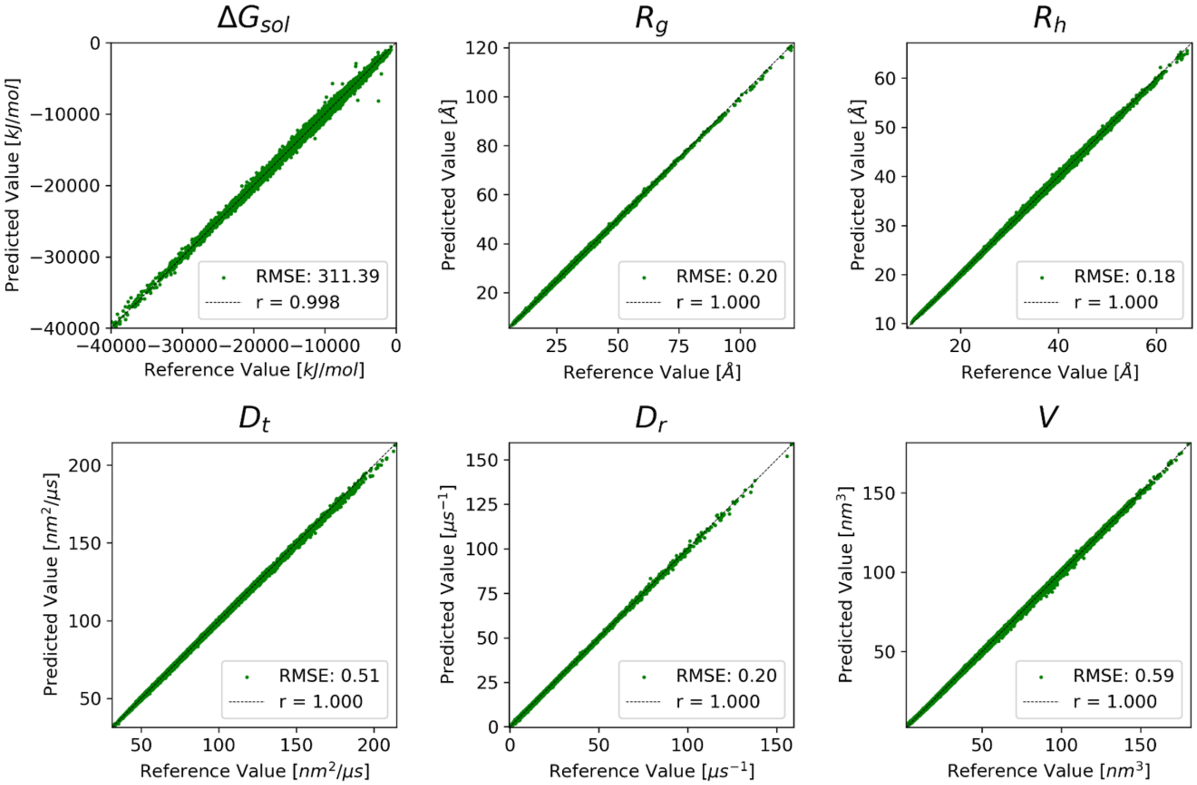

Figure S8. Training set predictions of transformer model for global molecular properties vs. calculated reference values. Results are shown for ΔG_{sol}, R_g, R_h, D_t, D_r, and V. RMSE values are given in units as displayed on the axes of each subplot.

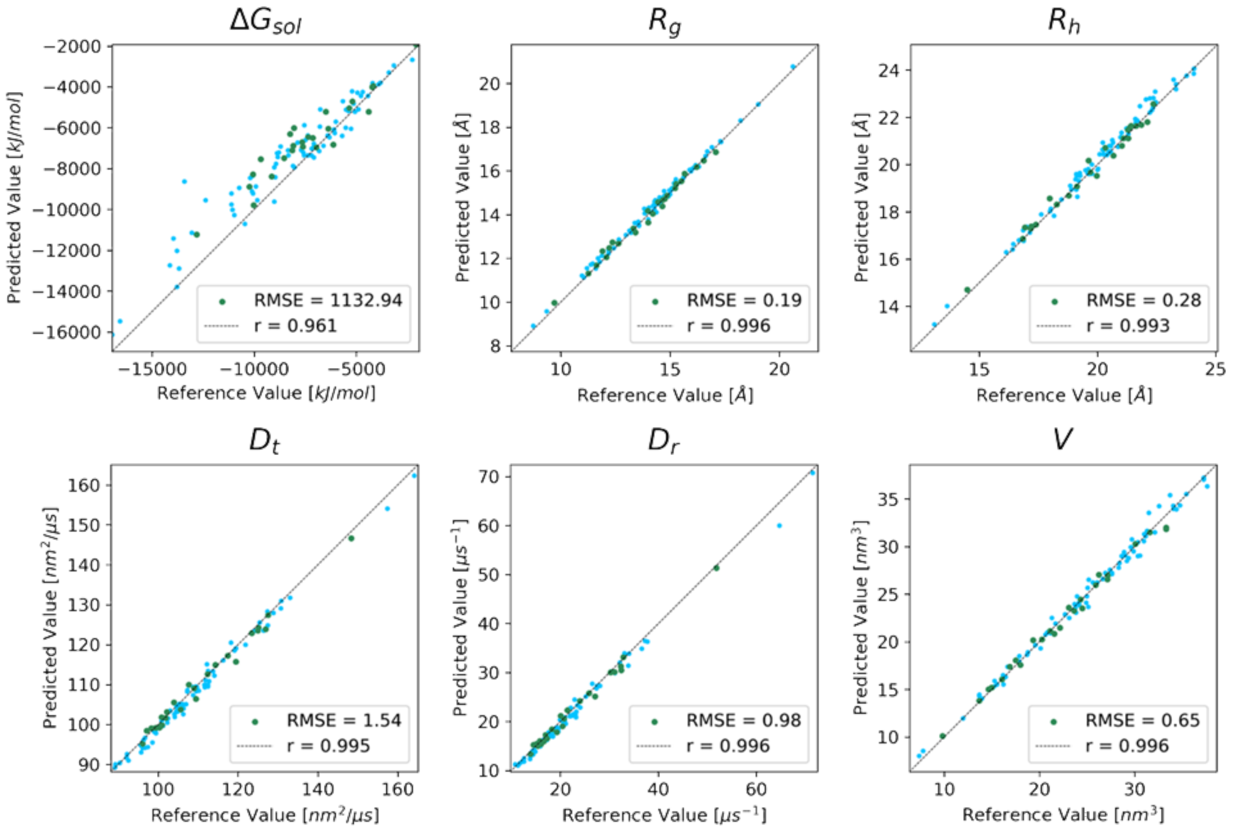

Figure S9. GSnet performance for predictions of global molecular properties on small protein test set. Predictions are shown for 123 dissimilar (<20% identity), small (40-200 residues) PDB structures. (chosen via PISCES[6]; resolution < 1.5 Å, R<0.25). Darker green points represent structures that have low fold-level similarity (TM-score < 0.6) to any of the structures in the training set. RMSE values are given in units as displayed on the axes of each subplot, for all datapoints on the plots.

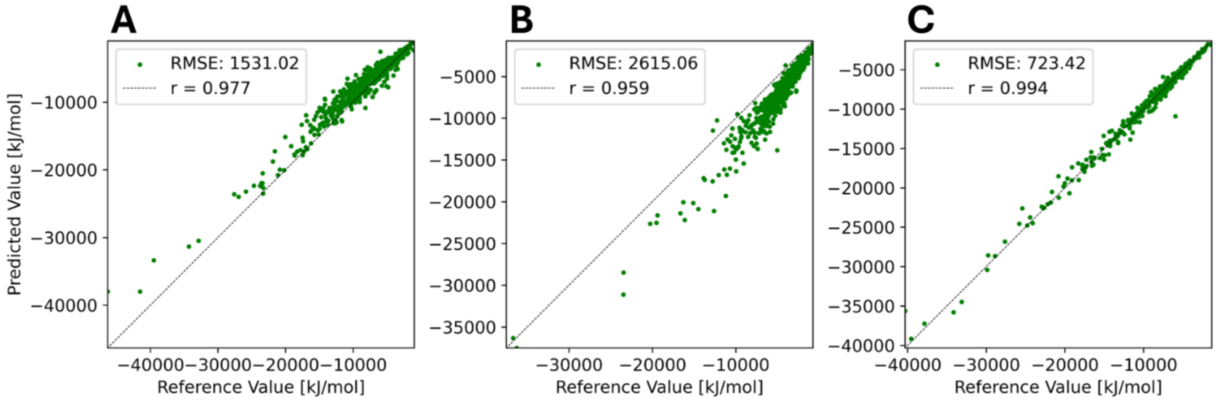

Figure S10. GSnet performance for predictions of ΔG_{sol} on extended PDB test set. Predictions are shown for 610 proteins without (A) and with (B) energy minimization. Results with AlphaFold2[7] models (C) are shown for comparison. RMSE values are given in kJ/mol.

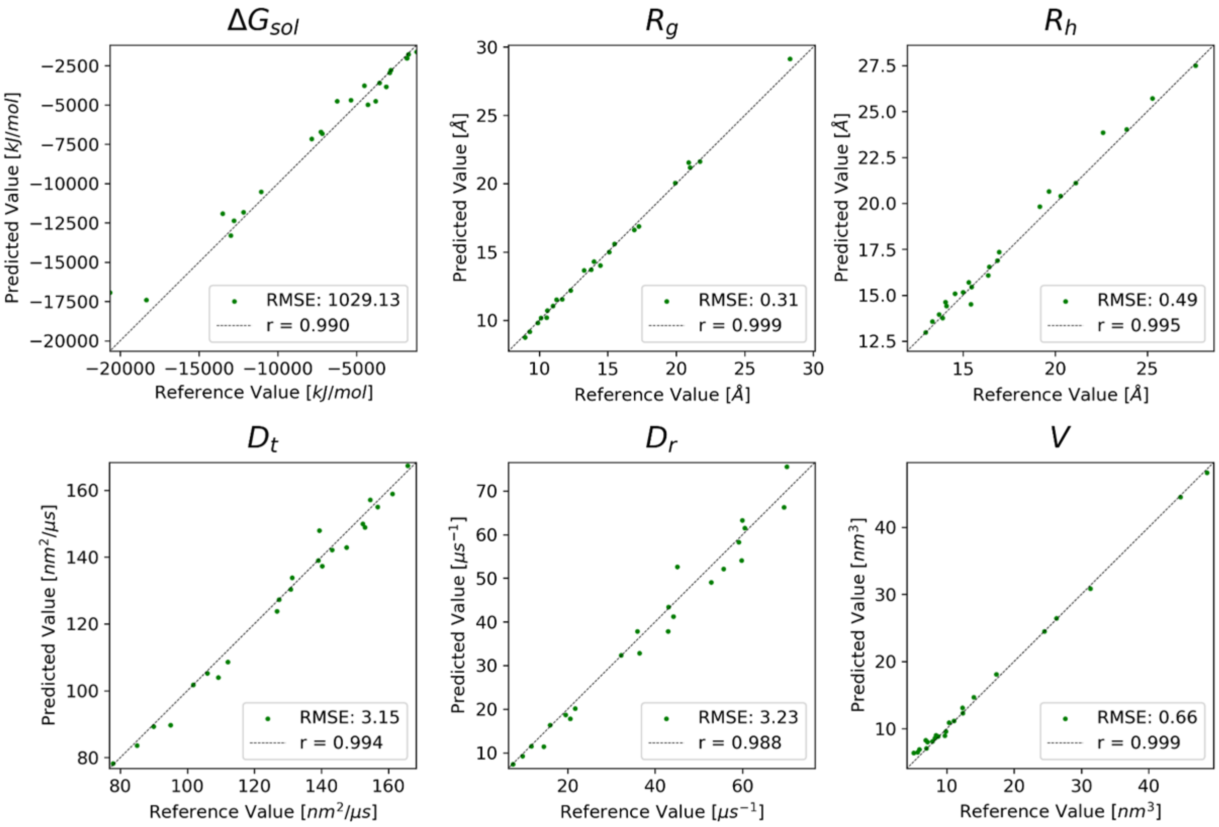

Figure S11. GSnet performance for predictions of global molecular properties for orphan proteins. Predictions are shown for 23 of the 25 orphan proteins contained in the Orphan25 dataset[8] for which experimental PDB structures were available without missing and/or non-standard residues. RMSE values are given in units as displayed on the axes of each subplot.

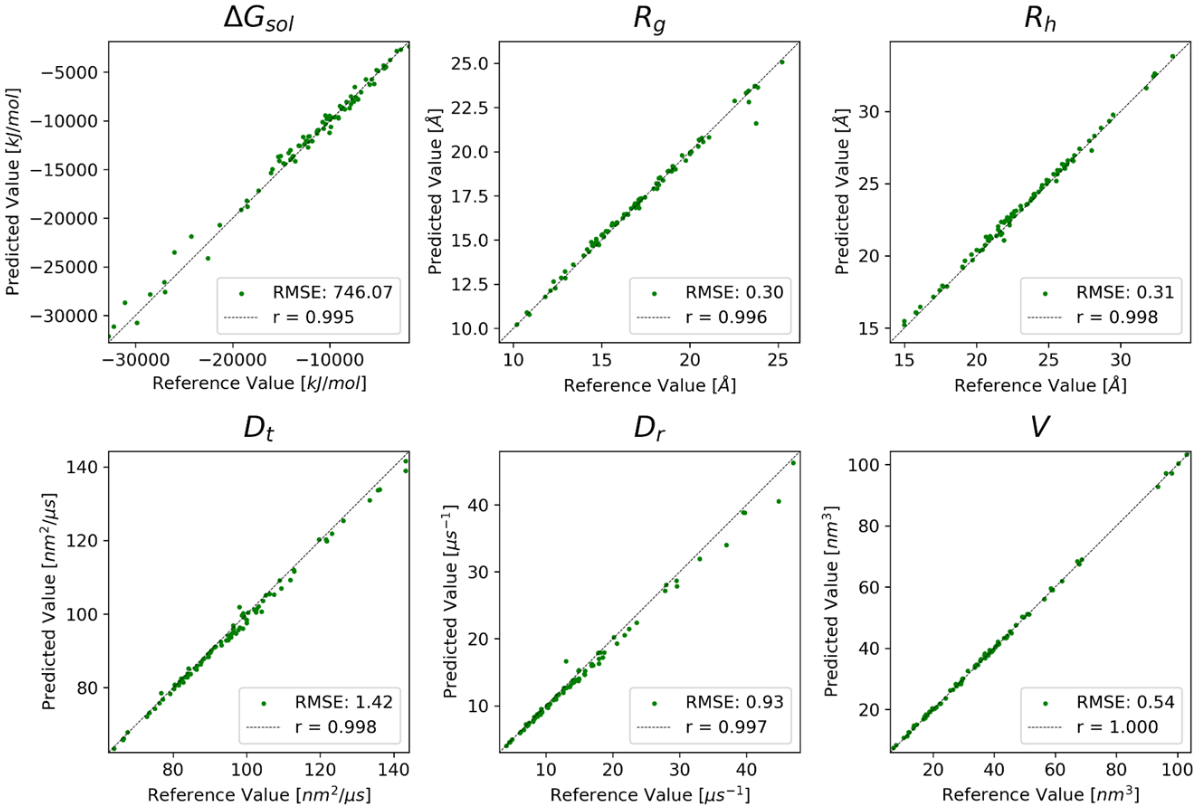

Figure S12. GSnet predictions for a subset of 100 ESMFold-predicted structures out of the original training set. RMSE values are given in units as displayed on the axes of each subplot.

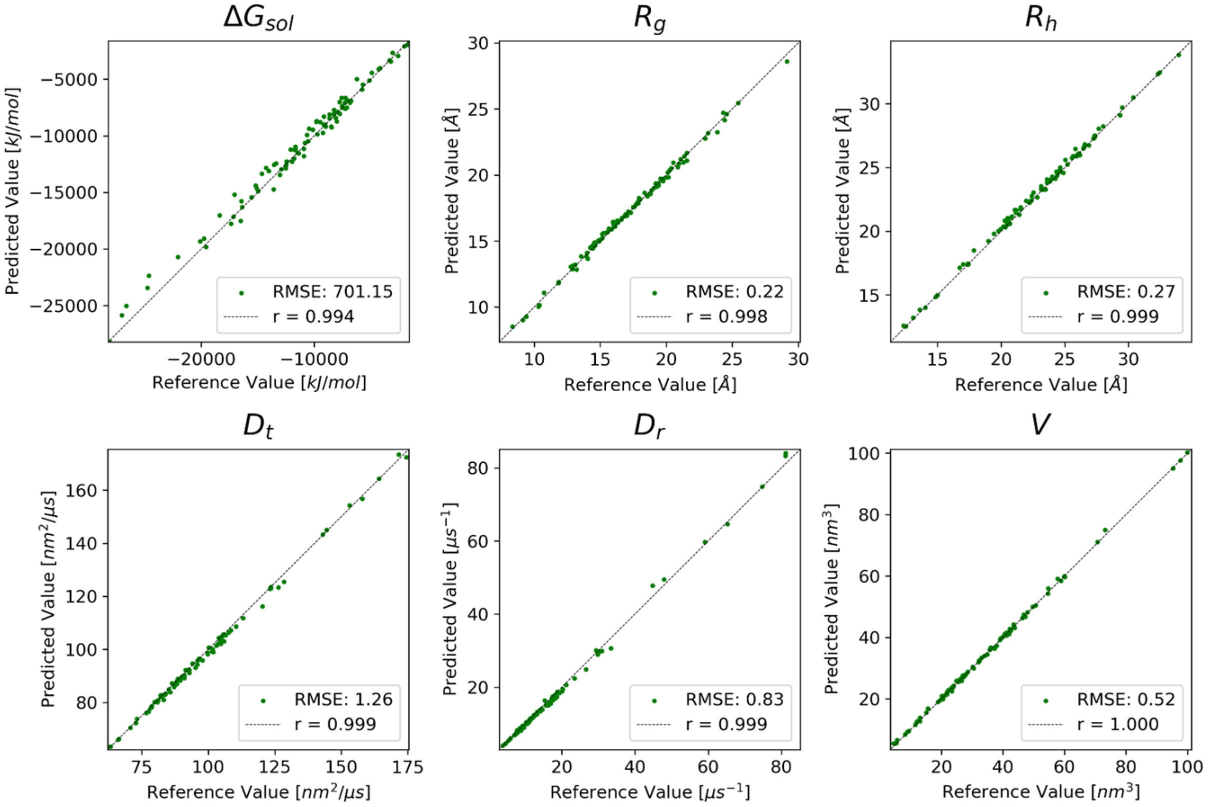

Figure S13. GSnet predictions for a subset of 100 ESMFold-predicted structures out of the original validation set. RMSE values are given in units as displayed on the axes of each subplot.

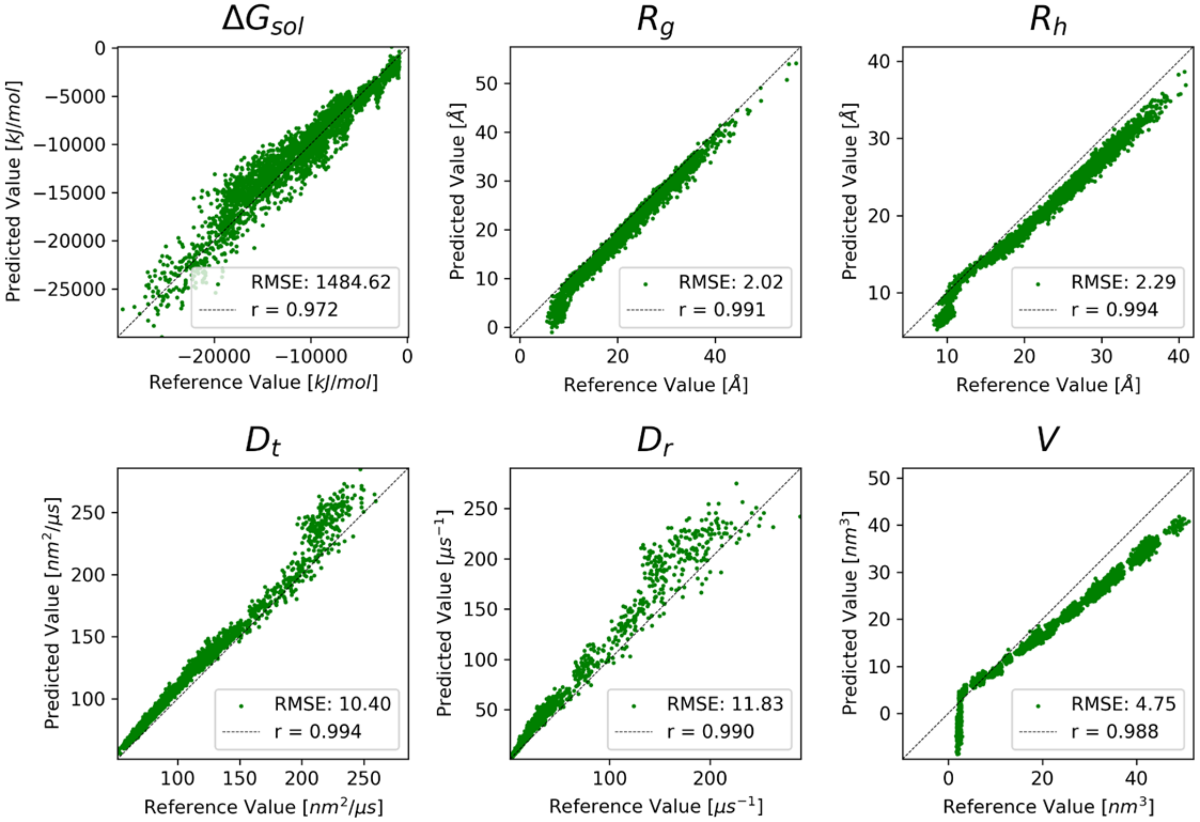

Figure S14. GSnet performance on IDP structures. This validation set consists of 4,500 total IDP structures (ensembles of 100 structures for 45 distinct peptides; generated via *cg2aa*). RMSE values are given in units as displayed on the axes of each subplot.

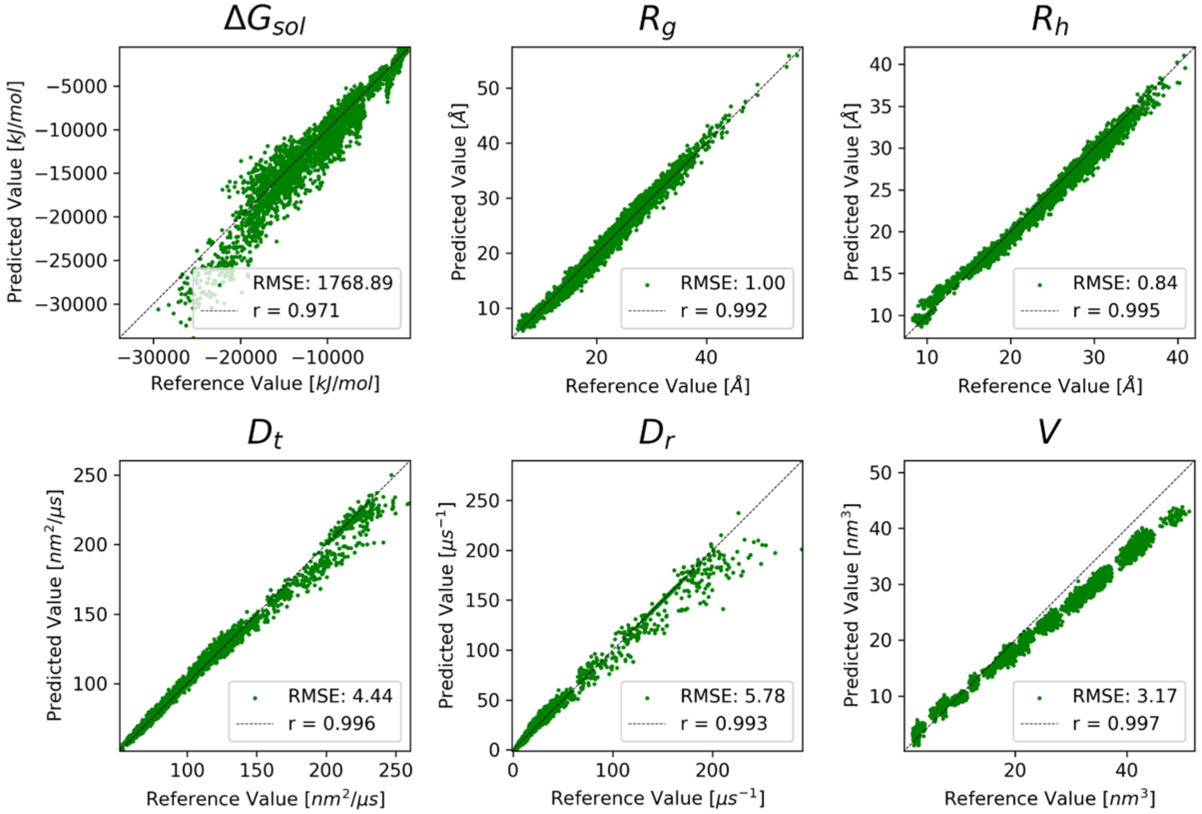

Figure S15. Retrained GSnet performance on IDP structures. Performance of GSnet after fine-tuning on a training set consisting of 100 angiotensin structures. The validation set consists of 4,500 total IDP structures (ensembles of 100 structures for 45 distinct peptides; generated via *cg2aa*). RMSE values are given in units as displayed on the axes of each subplot.

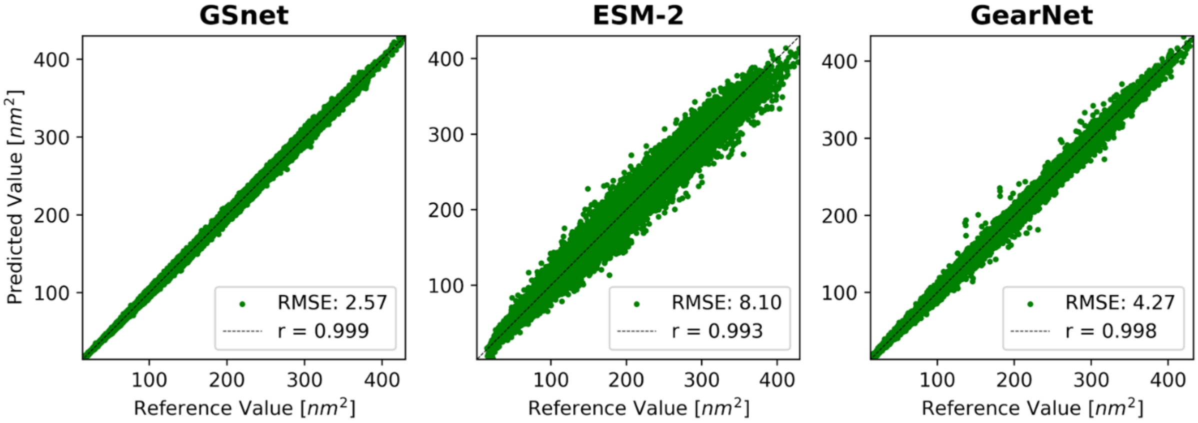

Figure S16. Training set performance of embedding-based SASA predictors. Correlation coefficients from linear fits are shown in the upper left corner of each plot. RMSE values are given in nm^2.

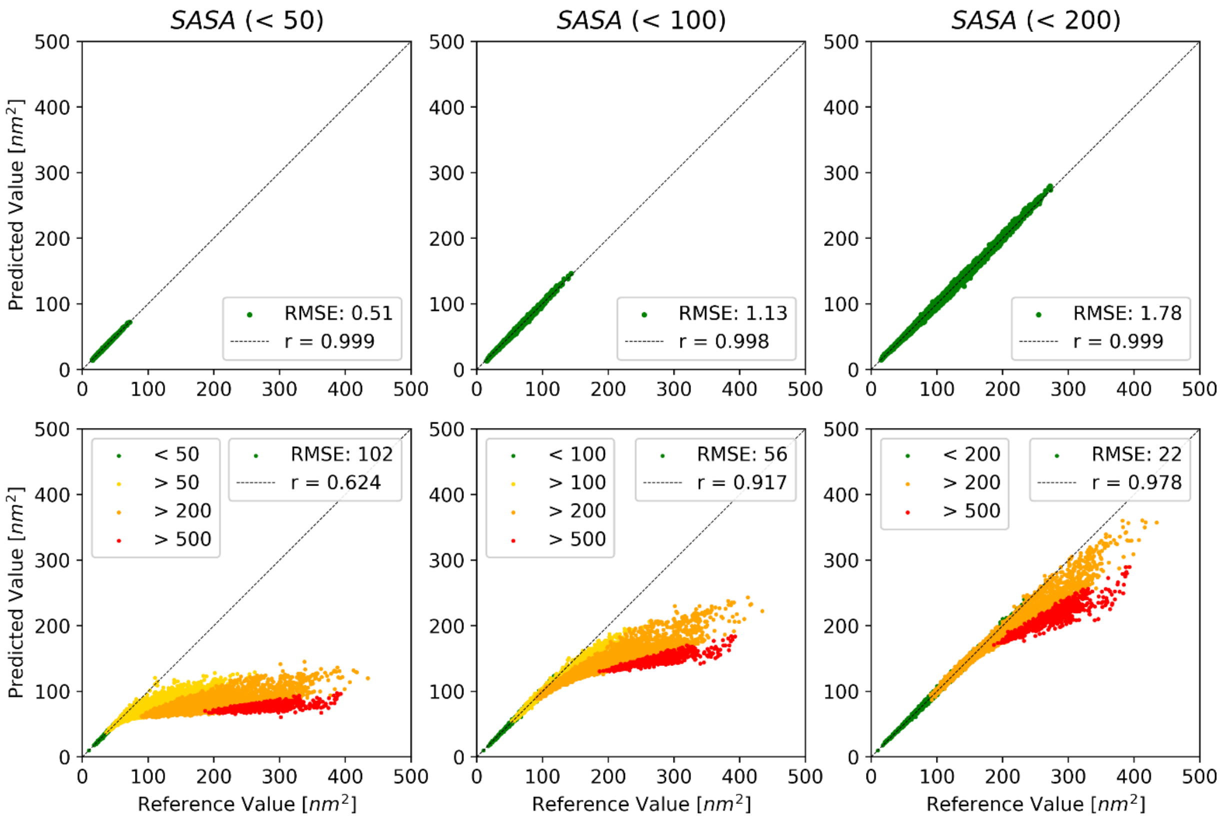

Figure S17. GSnet-based SASA predictors trained on proteins with limited size. Model performance is shown for training (top row) and validation (bottom row) sets. Numbers in parentheses indicate maximum size (in residues) of structures in training set. Each data point represents one protein, with colors signifying the protein size as indicated in the figure legend. Pearson correlation coefficients and RMSEs encompass all data shown in the plots. RMSE values are given in nm^2.

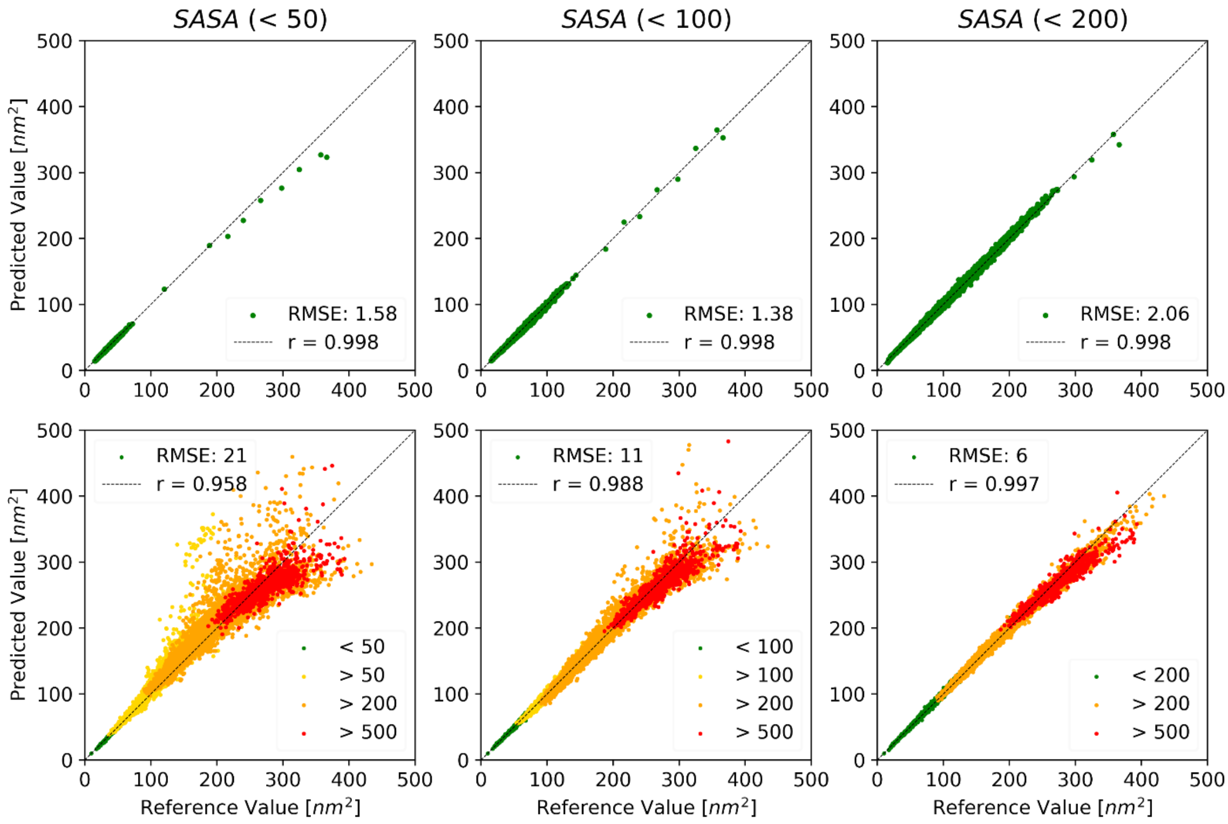

Figure S18. GSnet-based SASA predictors trained on proteins with limited size and augmented data for larger proteins. Model performance is shown for training (a) and validation (b) sets. Numbers in parentheses indicate maximum size (in residues) of structures in training set, excluding augmented data points. Each data point represents one protein, with colors signifying the protein size as indicated in the figure legend. Pearson correlation coefficients and RMSEs encompass all data shown in the plots. RMSE values are given in nm^2.

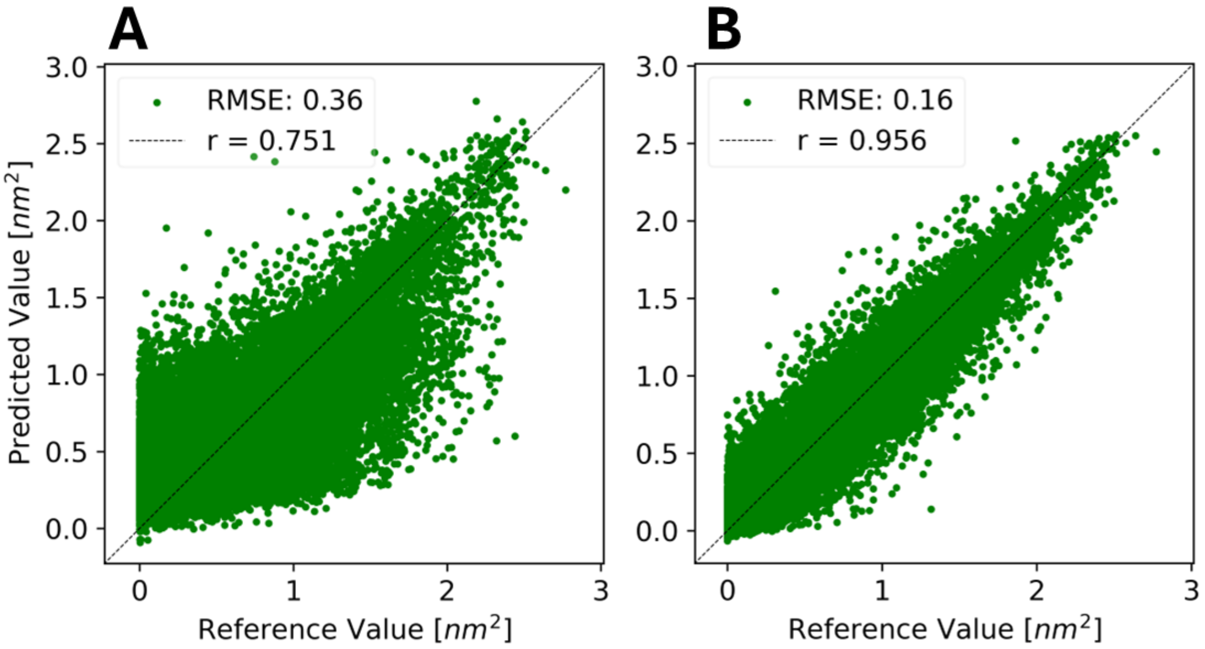

Figure S19. GSnet-based residue-level SASA predictions. Plots display model predictions on the validation set vs. calculated reference values when using: (A) a fixed, pretrained GNN where only the output MLP was trained, and (B) a pretrained GNN where both the GNN parameters and the output MLP were optimized during training. Correlation coefficients from linear fits and RMSE values are shown in the lower right corner of each plot. RMSE values are given in nm^2.

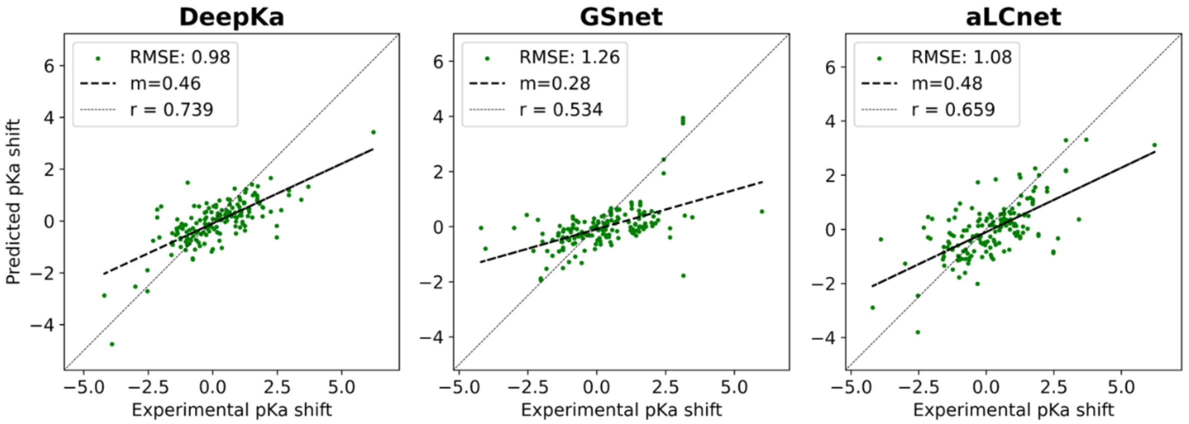

Figure S20. Experimental pKa shift predictions for EXP67S test set with DeepKa, GSnet, and aLCnet. For GSnet and aLCnet models the test performance is shown for the model with the lowest validation RMSE (out of 100 trained models). Performance data for DeepKa was obtained via predictions made with the DeepKa Web Server[9]. RMSE values are given in p*K* units.

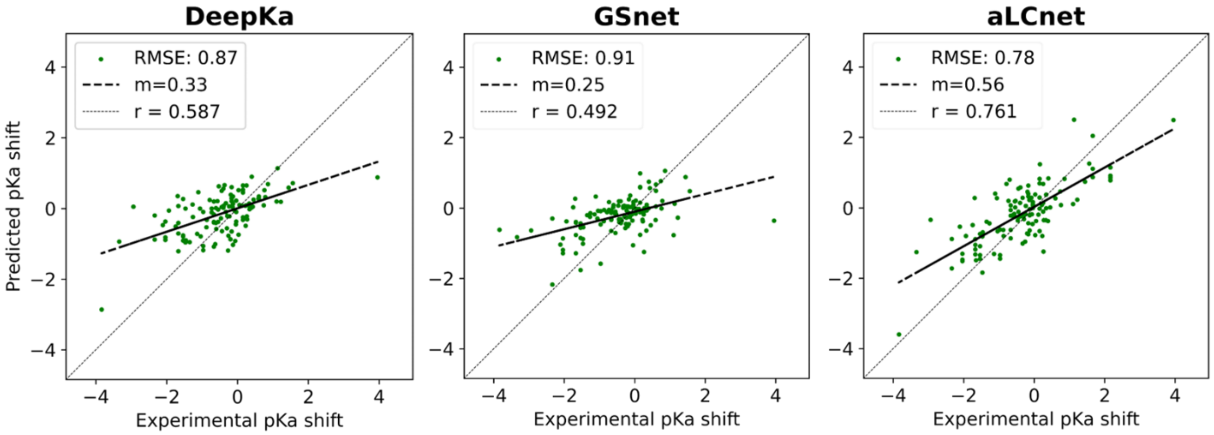

Figure S21. Experimental pKa shift predictions with DeepKa, GSnet, and aLCnet on MSU-pKa-test, with datapoints corresponding to CYS and TYR residues removed. For GSnet and aLCnet models the test performance is shown for the model with the lowest validation RMSE (out of 20 trained models). Performance data for DeepKa were obtained via predictions made with the DeepKa web server[9]. RMSE values are given in pK units. Predictions were made using experimental PDB structures.

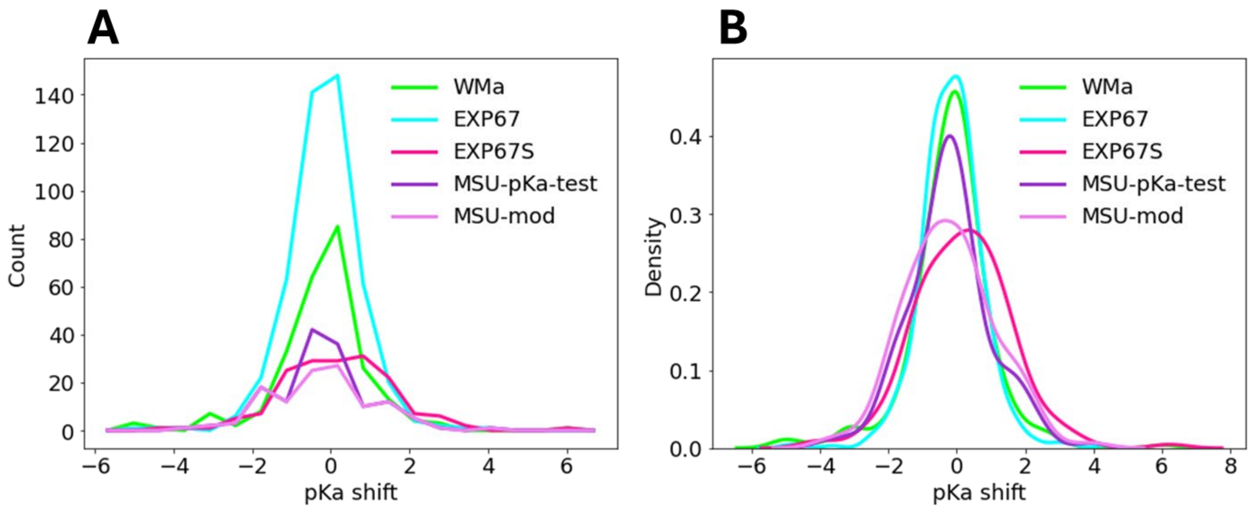

Figure S22. Absolute (A) and relative (B) pKa shift distributions for various pKa test sets. "MSU-mod" refers to a modified version of the MSU-pKa-test set in which datapoints with shifts closest to the mean were removed until the standard deviation of the set was within 0.1 p<u>*K*</u> units of the standard deviation of EXP67s.

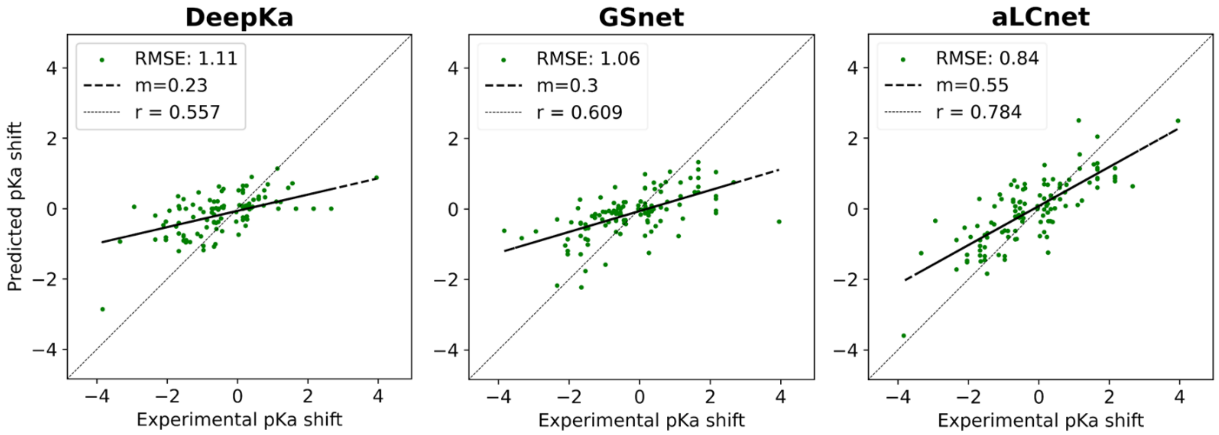

Figure S23. Experimental pKa shift predictions with DeepKa, GSnet, and aLCnet on a subset of MSU-pKa-test. MSU-pKa-test was modified such that datapoints with shifts closest to the mean were removed until the standard deviation of the set was within 0.1 p<u>K</u> units of the standard deviation of EXP67s. For GSnet and aLCnet models the test performance is shown for the model with the lowest validation RMSE (out of 20 trained models). Performance data for DeepKa were obtained via predictions made with the DeepKa web server[9]. DeepKa predicted shifts for tyrosine and cysteine residues were obtained with the null model. RMSE values are given in pK units. Predictions were made using experimental PDB structures.

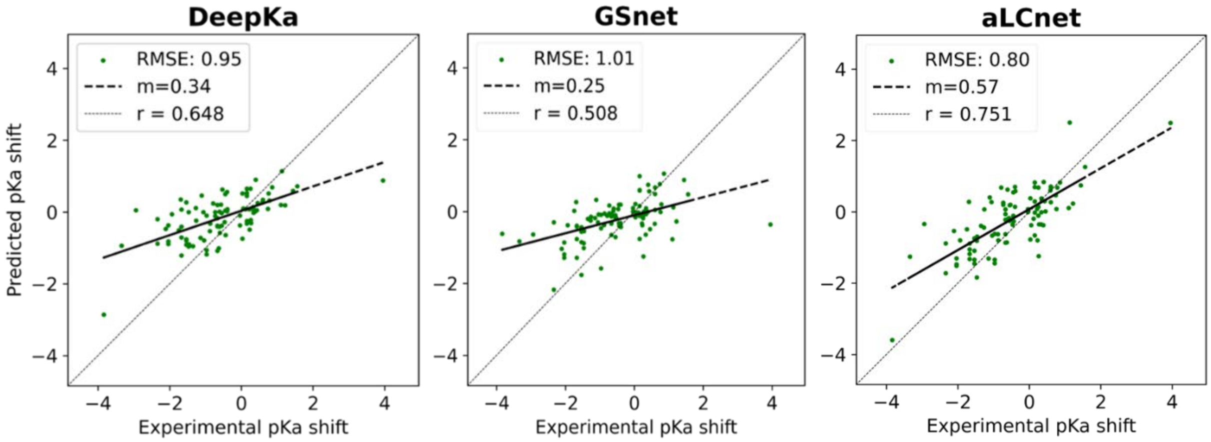

Figure S24. Experimental pKa shift predictions with DeepKa, GSnet, and aLCnet on a subset of MSU-pKa-test, with datapoints corresponding to CYS and TYR residues removed. MSU-pKa-test was modified such that datapoints with shifts closest to the mean were removed until the standard deviation of the set was within 0.1 p$\underline{K}$ units of the standard deviation of EXP67s. For GSnet and aLCnet models the test performance is shown for the model with the lowest validation RMSE (out of 20 trained models). Performance data for DeepKa were obtained via predictions made with the DeepKa web server[9]. RMSE values are given in pK units. Predictions were made using experimental PDB structures.

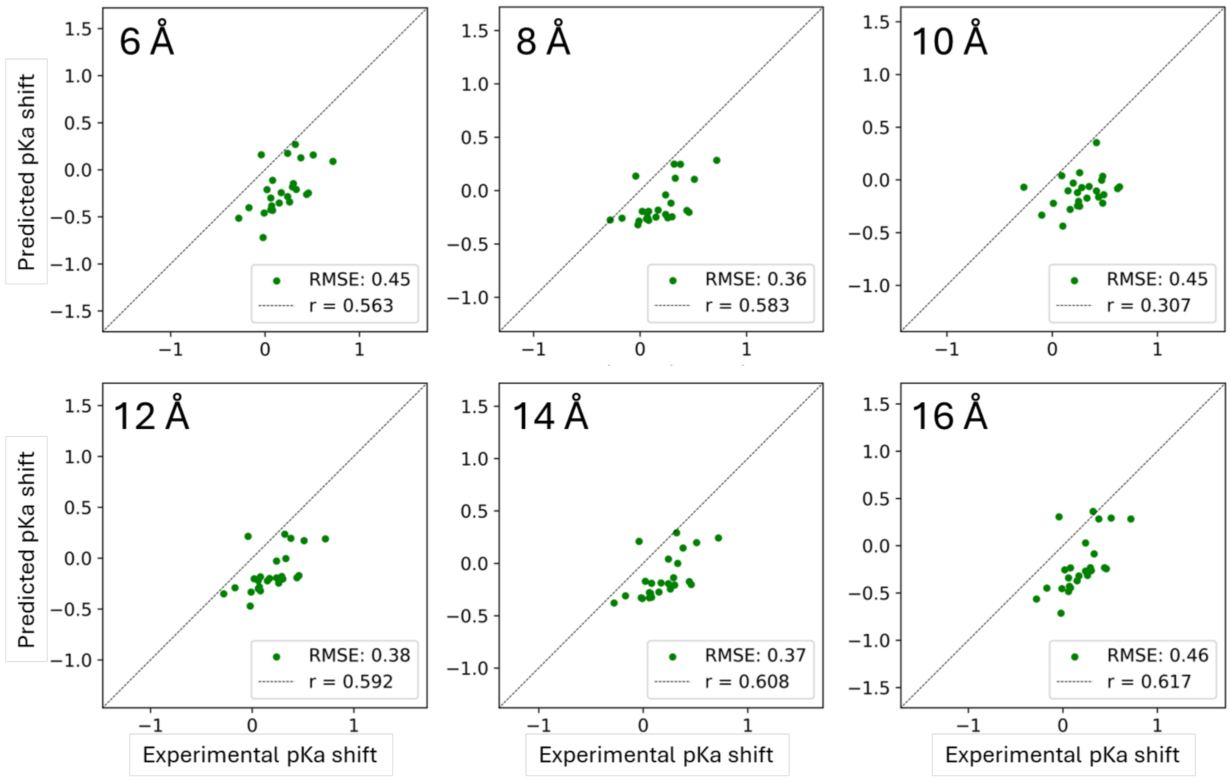

Figure S25. CHARMM-based aLCnet predictions of pK_a shifts for selected residues in α-synuclein with various selection cutoffs ranging from six to sixteen angstroms. Experimental values were obtained via NMR in 150 mM NaCl by Croke *et al.*[10]. RMSE values are given in pK units. Predictions were made over an ensemble of 300 simulated structures and averaged.

Table S1. Input features to GSnet.

**Feature** [Shape]	**Description**
**x** $[N, 3]$	Cartesian coordinates of C_α atoms.
**aa** $[N, 20]$	Integer encoding of amino acid sequence.
**dh** $[N, 15]$	Two backbone dihedral angles (φ,ψ) and the first three side-chain dihedral angles (χ_1, χ_2, χ_3) represented as mask, cosine & sine encoding.
**cc** $[N, 1]$	Distances from C_α atoms to protein center of mass.

N is the number of residues in the input protein

Table S2. Input features to aLCnet.

**Feature** [Shape]	**Description**
**x** $[N, 3]$	Cartesian coordinates of heavy atoms.
**aa** $[N, 20]$	Integer encoding of amino acid type.
**atom** $[N, 4]$	Integer encoding of atom type.
**charge** $[N, 1]$	Effective charge of atom as computed by PDB2PQR.

N is the number of atoms in the input selection

Table S3. Graph features used as input to MLP. Features vary according to the predicted properties and are extracted after message passing. "Pretrain" refers to the original training on ΔG_{sol}, R_g, R_h, D_t, D_r, V. "SASA" refers to fine-tuning for molecular-level SASA, "rSASA" refers to fine-tuning for residue-level SASA, and "pK_a" refers to training on pK_a values. d is the number of hidden dimensions of the GNN, N is the number of features extracted with dimensionality d, and $d \cdot N$ is the number of input channels to the MLP.

Model	Task	MLP input Features	N	$d \cdot N$
**GSnet** $d = 150$	Pretrain	$\boldsymbol{\mu}$	1	150
	SASA	$\boldsymbol{\mu}$	1	150
	rSASA	$\mathbf{h}_i$	1	150
	pK_a	concat($\mathbf{h}_i, \boldsymbol{\mu}_6, \boldsymbol{\mu}_8, \boldsymbol{\mu}_{10}, \boldsymbol{\mu}_{12}, \boldsymbol{\mu}_{15}, \boldsymbol{\mu}$)	7	1,050
**aLCnet** $d = 75$	pK_a	concat($\mathbf{h}_{C\alpha}, \boldsymbol{\mu}_{aa}, \boldsymbol{\mu}$)	3	225

Table S4. Training set performance for prediction of global molecular properties. Mean absolute percent errors (MAPE) and root mean square errors (RMSE) are reported as the mean and standard errors from 20 independent training runs (except for ESM-2).

	**Model**	**ΔG_{sol} [kJ/mol]**	**R_g [Å]**	**R_h [Å]**	**D_t [nm^2/μs]**	**D_r [$μs^{-1}$]**	**V [nm^3]**
**MAPE (%)**	*Transformer (GSnet)*	3.29 ± 0.09	0.72 ± 0.02	0.53 ± 0.01	0.53 ± 0.01	2.10 ± 0.04	1.04 ± 0.01
	EGNN	2.81 ± 0.07	0.76 ± 0.02	0.50 ± 0.01	0.51 ± 0.01	2.05 ± 0.04	1.03 ± 0.01
	SchNet	2.03 ± 0.04	0.71 ± 0.01	0.41 ± 0.01	0.41 ± 0.01	1.89 ± 0.04	1.02 ± 0.01
	GearNet	3.79 ± 0.05	1.57 ± 0.01	0.98 ± 0.01	0.98 ± 0.01	3.85 ± 0.04	2.50 ± 0.02
	ESM-2[a]	8.64	4.60	2.65	2.65	4.60	5.12
**RMSE**	*Transformer (GSnet)*	393.6 ± 11.7	0.25 ± 0.01	0.21 ± 0.01	0.55 ± 0.01	0.23 ± 0.01	0.61 ± 0.01
	EGNN	336.2 ± 9.1	0.25 ± 0.01	0.20 ± 0.01	0.53 ± 0.01	0.22 ± 0.00	0.58 ± 0.00
	SchNet	231.7 ± 3.6	0.22 ± 0.00	0.15 ± 0.00	0.43 ± 0.01	0.21 ± 0.00	0.58 ± 0.01
	GearNet	456.4 ± 4.5	0.54 ± 0.01	0.39 ± 0.00	0.96 ± 0.01	0.38 ± 0.01	1.67 ± 0.01
	ESM-2[a]	1145.0	1.85	1.20	2.91	1.36	3.46

[a]Only one model was trained with ESM-2 due to computational cost.

Table S5. Performance metrics for aLCnet with varying edge cutoffs when evaluated on the PHMD549 validation set. Metrics averaged over 5 training runs. Performance results are given as mean ± SEM with the best (out of 5) result given in parentheses.

Edge Cutoff Distance [Å]	RMSE	R	m
**5.0**	0.79 ± 0.00 (0.79)	0.65 ± 0.00 (0.66)	0.43 ± 0.01 (0.49)
**6.0**	0.83 ± 0.01 (0.79)	0.61 ± 0.01 (0.65)	0.37 ± 0.01 (0.42)
**7.0**	0.85 ± 0.00 (0.84)	0.59 ± 0.00 (0.61)	0.33 ± 0.01 (0.37)
**8.0**	0.87 ± 0.00 (0.85)	0.55 ± 0.00 (0.58)	0.28 ± 0.01 (0.33)

Table S6. Performance metrics for aLCnet with varying maximum number of edges per node with an edge cutoff of 5 Å when evaluated on the PHMD549 validation set. Metrics averaged over 5 training runs. Performance results are given as mean ± SEM with the best (out of 5) result given in parentheses.

Max Edges Per Node	RMSE	R	m
**50**	0.87 ± 0.00 (0.86)	0.55 ± 0.00 (0.57)	0.31 ± 0.00 (0.34)
**75**	0.85 ± 0.00 (0.84)	0.58 ± 0.00 (0.59)	0.34 ± 0.00 (0.35)
**100**	0.79 ± 0.00 (0.79)	0.65 ± 0.00 (0.66)	0.43 ± 0.01 (0.49)
**150**	**0.78 ± 0.00 (0.76)**	**0.67 ± 0.00 (0.69)**	**0.44 ± 0.00 (0.47)**
**200**	0.79 ± 0.00 (0.77)	0.66 ± 0.00 (0.68)	0.41 ± 0.01 (0.48)
**250**	0.79 ± 0.00 (0.76)	0.67 ± 0.00 (0.69)	0.43 ± 0.01 (0.49)
**300**	0.81 ± 0.00 (0.79)	0.64 ± 0.00 (0.66)	0.38 ± 0.01 (0.45)

Table S7. Performance metrics for CHARMM-based aLCnet with varying locality when evaluated on MSU-pKa-test. Metrics averaged over 5 training runs. Performance results are given as mean ± SEM with the best (out of 5) result given in parentheses.

Cutoff Distance [Å]	RMSE	R	m
**6.0**	0.99 ± 0.00 (0.97)	0.58 ± 0.01 (0.62)	0.25 ± 0.00 (0.28)
**8.0**	1.01 ± 0.01 (0.95)	0.55 ± 0.01 (0.62)	0.27 ± 0.01 (0.34)
**10.0**	**0.95 ± 0.00 (0.89)**	**0.62 ± 0.00 (0.68)**	**0.38 ± 0.01 (0.59)**
**12.0**	1.00 ± 0.01 (0.94)	0.60 ± 0.01 (0.64)	0.37 ± 0.02 (0.60)
**14.0**	0.98 ± 0.01 (0.96)	0.59 ± 0.01 (0.62)	0.31 ± 0.01 (0.36)
**16.0**	0.99 ± 0.01 (0.88)	0.59 ± 0.01 (0.67)	0.32 ± 0.01 (0.45)

Table S8. Test RMSE by residue type for the various pKa prediction methods. GearNet, ESM, GSnet and aLCnet models selected based on lowest validation RMSE.

**Model**	ASP	GLU	HIS	LYS	TYR	CYS
Null Model	1.13	0.58	1.83	0.51	1.72	1.65
PROPKA	0.91	0.64	1.22	0.50	0.82	4.10
GSnet	1.22	0.63	0.74	0.94	1.11	0.11
aLCnet	0.94	0.72	1.15	0.50	1.08	1.03

Table S9. Computational cost of different methods for predicting global molecular properties via GSnet. HYDROPRO estimates hydrodynamic properties R_g, R_h, D_t, D_r, and V. APBS estimates electrostatic solvation free energies ΔG_{sol}. GSnet estimates all properties at once. Structures were obtained from the AlphaFold Protein Structure Database[11], and UNIPROT codes are provided in the table. For GSnet, "Program" represents the total time it takes the program to run, including loading packages, loading models, and converting data, while "Forward" represents the time it takes for a forward pass of the model, which is more relevant for high-throughput predictions. Timings were obtained via the 'time' command in Linux, and via the time module in Python for forward passes of GSnet.

	**UNIPROT**	**Residues**	**HYDROPRO**	**APBS**	**GSnet**[a]	
					Program	Forward
**Wall Clock Time [s]**	P80967	50	6.0	196.8	3.25	1.09
	Q16878	200	25.0	469.9	3.46	1.11
	P80404	500	244.4	2712.8	3.92	1.17
**User CPU Time [s]**	P80967	50	69.1	188.4	19.83	--
	Q16878	200	354.4	459.9	26.17	--
	P80404	500	4110	2687.3	42.72	--
**Peak memory usage [GB]**	P80967	50	0.11	11.17	0.478	--
	Q16878	200	0.33	12.20	0.532	--
	P80404	500	1.73	30.80	0.670	--

[a] Statistics averaged over 10 runs.

Supplemental References

(1) Chen, A. Y.; Lee, J.; Damjanovic, A.; Brooks, B. R. Protein pKa Prediction by Tree-Based Machine Learning. *J. Chem,. Theory Comp.* **2022**, *18*, 2673-2686.
(2) Gokcan, H.; Isayev, O. Prediction of Protein pKa with Representation Learning. *Chem. Sci.* **2022**, *13*, 2462-2474.
(3) Wilson, C. J.; Karttunen, M.; de Groot, B. L.; Gapsys, V. Accurately Predicting Protein pKa Values Using Nonequilibrium Alchemy. *J. Chem,. Theory Comp.* **2023**, *19*, 7833-7845.
(4) Shi, Y.; Huang, Z.; Feng, S.; Zhong, H.; Wang, W.; Sun, Y. Masked label prediction: Unified message passing model for semi-supervised classification. *arXiv preprint* **2020**, arXiv:2009.03509. DOI: 10.48550/arXiv.2009.03509.
(5) Lei Ba, J.; Kiros, J. R.; Hinton, G. E. Layer normalization. *arXiv preprint* **2016**, arXiv:1607.06450. DOI: 10.48550/arXiv.1607.06450.
(6) Wang, G.; Dunbrack Jr, R. L. PISCES: a protein sequence culling server. *Bioinformatics* **2003**, *19*, 1589-1591.
(7) Jumper, J.; Evans, R.; Pritzel, A.; Green, T.; Figurnov, M.; Ronneberger, O.; Tunyasuvunakool, K.; Bates, R.; Žídek, A.; Potapenko, A. Highly accurate protein structure prediction with AlphaFold. *Nature* **2021**, *596*, 583-589.
(8) Wang, W.; Peng, Z.; Yang, J. Single-sequence protein structure prediction using supervised transformer protein language models. *Nat. Comp. Sci.* **2022**, *2*, 804-814.
(9) Cai, Z.; Peng, H.; Sun, S.; He, J.; Luo, F.; Huang, Y. DeepKa Web Server: High-Throughput Protein pKa Prediction. *J. Chem. Inf. Model.* **2024**, *64*, 2933-2940.
(10) Croke, R. L.; Patil, S. M.; Quevreaux, J.; Kendall, D. A.; Alexandrescu, A. T. NMR determination of pKa values in α-synuclein. *Prot. Sci.* **2011**, *20*, 256-269.
(11) Varadi, M.; Anyango, S.; Deshpande, M.; Nair, S.; Natassia, C.; Yordanova, G.; Yuan, D.; Stroe, O.; Wood, G.; Laydon, A.; et al. AlphaFold Protein Structure Database: massively expanding the structural coverage of protein-sequence space with high-accuracy models. *Nucl. Acids Res.* **2022**, *50*, D439-D444.
